# An Investigation of Metabolic Risk Factors and Gut Microbiota in Unexplained Syncope

**DOI:** 10.3390/biomedicines12020264

**Published:** 2024-01-24

**Authors:** Susanna Longo, Federica Del Chierico, Matteo Scanu, Francesca Toto, Jacopo M. Legramante, Stefano Rizza, Lorenza Putignani, Massimo Federici

**Affiliations:** 1 Department of Systems Medicine, University of Rome Tor Vergata, Via Montpellier 1, 00133 Rome, Italy; susanna.longo@uniroma2.it (S.L.); legraman@uniroma2.it (J.M.L.); rizza@med.uniroma2.it (S.R.); 2 Unit of Human Microbiome, Bambino Gesù Children’s Hospital, IRCCS, 00165 Rome, Italy; federica.delchierico@opbg.net (F.D.C.); matteo.scanu@opbg.net (M.S.); francesca.toto@opbg.net (F.T.); 3 Unit of Microbiomics and Unit of Human Microbiome, Bambino Gesù Children’s Hospital, IRCCS, 00165 Rome, Italy; lorenza.putignani@opbg.net

**Keywords:** unexplained syncope, gut microbiota, cardiometabolic risk factors, cardiovascular diseases

## Abstract

Background: The pathogenesis of many syncopal episodes remains unexplained. Intestinal dysbiosis could be involved in the pathophysiological mechanisms of syncope due to its connection with the central nervous system via the microbiota–gut–brain axis. This pilot study aimed to explore the specific cardiometabolic risk factors and gut microbiota in unexplained syncope (US), compared to other types of syncope, to assess their similarity or verify their different origins. Methods: We studied 86 participants with syncope, who were divided into four groups: an orthostatic syncope group (OH, *n* = 24), a neuromediated syncope group (NMS, *n* = 26), a cardiological syncope group (CS, *n* = 9), and an unexplained syncope group (US, *n* = 27). We evaluated the anthropometric, clinical, and metabolic characteristics of the four groups; the α- and β-diversity; and the differences in the abundance of the microbial taxa. Results: The US group had a lower incidence of systolic hypertension at the first visit and a lower frequency of patients with nocturnal hypertension than the CS group. Compared to the OH and NMS groups, the US group had a higher incidence of carotid plaques and greater carotid intima–media thickness, respectively. The microbiota differed significantly between the US and CS groups, but not between the US group and the OH or NMS group. Conclusions: We observed significant differences in the gut microbiota between CS and US. Future studies are necessary to evaluate the involvement of the gut microbiota in the complex pathogenesis of syncope and whether its analysis could support the interpretation of the pathophysiological mechasnisms underlying some episodes classifiable as US.

## 1. Introduction

Syncope is a transient loss of consciousness (TLC) due to cerebral hypoperfusion, which is characterized by amnesia, loss of postural tone, and occasional perturbation of smooth muscle control. Syncope has a rapid onset, short duration, and spontaneous resolution [1,2].

Although several hypotheses have been described, the pathogenesis of most syncope events remains to be elucidated [1], thus making diagnosis and risk stratification considerably difficult, particularly for events that cannot be clearly categorized.

According to the 2018 European Society of Cardiology (ESC) guidelines [2], three types of syncope exist: reflex or neurologically mediated syncope (NMS), which is associated with a specific trigger; syncope due to orthostatic hypotension (OH), defined by a decrease of >20 mmHg in systolic blood pressure (SBP) or >10 mmHg in diastolic blood pressure (SBP) after standing for 3 min; and cardiac syncope (CS), which is caused by arrhythmic pathologies or structural diseases of the heart and great vessels. However, in clinical practice, syncope commonly shows characteristics that cannot be clearly classified according to the ESC criteria, a condition defined as unexplained syncope (US).

According to the Framingham Heart Study, the incidence of syncope in the past three decades of the last century was 6.2 per 1000 person-years, and the most frequent type was NMS (21.2% of cases), followed by CS and OH (9.5% and 9.4% of cases, respectively). The incidence of US was approximately 36.6% [3]. US often implies disorders with a poor prognosis. Several studies have demonstrated that US is a risk factor for sudden cardiac death [4]. Hence, the American College of Cardiology Foundation/American Heart Association guidelines (class IIa) and the ESC guidelines [2,5] consider the occurrence of US in individuals in the evaluation of implantable cardioverter-defibrillator placement. However, whether US is a subclinical expression of CS that must be reclassified later or whether US is a separate pathological entity with pathophysiological features that are more similar to those of NMS and OH remains unclear.

For the correct interpretation of US, it may be necessary to use new diagnostic strategies and new biomarkers. A link between the gut microbiota and vasovagal syncope has recently been proposed [6]. The contribution of intestinal dysbiosis to the pathophysiology of syncope seems to be supported by the role that the microbiota–gut–brain axis has in the development of cardiometabolic disorders and neurological pathologies [7].

This pilot study aimed to explore the specific cardiometabolic risk factors and gut microbiota in US, compared with other types of syncope, to assess their similarity or verify their different origins.

## 2. Materials and Methods

### 2.1. Patients

In this cross-sectional pilot study, we evaluated 104 patients treated at the Day Hospital service at the Tor Vergata University Hospital (Via Montpellier 1, 00133 Rome, Italy) between February 2020 and May 2022. All patients were at low risk of adverse events, according to the ESC risk stratification [2]; thus, they were referred to the Day Hospital service to continue the diagnostic process of syncope after being discharged from the Emergency Department. Patients were enrolled consecutively. The inclusion criteria were the presence of low-risk TLC according to ESC 2018 risk stratification [2]; age of 25–89 years; body mass index (BMI) > 18; good compliance with the research protocol; and ability to independently understand and sign informed consent forms. The exclusion criteria were pregnancy or breastfeeding; major psychiatric disorders (e.g., bipolar disorder, schizophrenia, schizoaffective disorder, psychosis, anorexia or bulimia nervosa, and obsessive–compulsive disorder); stage IV renal failure (indicated by eGFR < 30 mL/min); liver disease or liver failure (indicated by abnormal values of parameters such as alanine transaminase, aspartate aminotransferase, gamma-glutamyl transferase, alkaline phosphatase, or blood bilirubin > 5 times the upper reference value); donation of blood or blood products (>450 mL of plasma or platelets) immediately before the start of the study or after 4–12 weeks; participation in other study protocols in the 4 weeks before the provision of informed consent; and Mini-Mental State Examination cognitive assessment score < 24, corrected for age.

This study conformed to the principles outlined in the Declaration of Helsinki and was approved by the Bioethics Committee of the University of Rome Tor Vergata (study protocol trial register 57.21).

### 2.2. Assessment of Clinical and Metabolic Parameters

A complete clinical history was recorded regarding lifestyle habits, history of previous episodes of TLC, and presence of comorbidities (e.g., diabetes, cardiovascular disease, and arterial hypertension). Participants who regularly smoked at least one cigarette per day were counted as current smokers, and current smokers and former smokers were counted together in a single group, which was compared with non-smokers. Alcohol consumption was recorded as the number of drinks per day. Participants who engaged in physical activity for at least 1 h per week were considered physically active. The following clinical and metabolic anthropometric variables were measured: height, weight, waist circumference, random blood pressure (BP), and BMI (weight in kilograms divided by the square of height in meters). BP was measured in the dominant arm in a seated position using a standard sphygmomanometer cuff of an appropriate size. The presence of orthostatic hypotension, defined as an orthostatic decrease in SBP of 20 mmHg or DBP of 10 mmHg in normotensive participants, or a decrease in SBP of 30 mmHg in hypertensive participants, was assessed during the active standing test. Each patient was administered the Pittsburgh Sleep Quality Index questionnaire to assess sleep quality. Samples of blood, feces, and urine were also collected from each patient. Approximately 30 mL of whole blood was drawn between 8:00 and 9:00 a.m. after an overnight fast and used to perform routine laboratory evaluations, including complete blood count, total cholesterol, HDL cholesterol, triglycerides, eGFR, and glycemia.

### 2.3. Instrumental Exams

Carotid intima–media thickness (c-IMT) was calculated using the Esaote Mylab system (Ref 101620000) with a VF 13 × 10^−5^ linear-array transducer. Anterior, lateral, and posterolateral views were used to visualize the right and left common carotid arteries longitudinally. At each projection, three determinations of c-IMT were performed at 2 cm proximal to the bulb at the site of greatest thickness. The values at each site were averaged.

Non-invasive ambulatory 24 h BP monitoring was performed using a validated oscillometric recorder (TM-2430, A&D Instruments Ltd., Abingdon, UK) during a routine day and night setting. BP measurements were obtained every 15 min during the day and every 30 min during the night. The mean SBP and DBP for the day and night periods were calculated based on patient-reported data. Pattern dipping was expressed as the percentage difference between the mean daytime and nighttime pressure (Δ). The non-dipping and reverse-dipping patterns were indicated by a difference of ≤10% and ≤0%, respectively.

### 2.4. Collection of Fecal Samples and Pre-Processing of Fastq Files with QIIME2

Stool samples were collected using sterile disposable forceps from all participants in the morning after an overnight fast. Each fecal sample was immediately placed in a 5.0 mL Eppendorf Tube^®^ and stored at −80 °C until DNA extraction. Nucleic acid was extracted from each fecal sample (approximately 200 mg) using a QIAamp DNA Stool Mini Kit (Qiagen, Hilden, Germany). The obtained DNA was quantified using a NanoDrop™ 2000/2000c spectrophotometer (Thermo Fisher Scientific, Waltham, MA, USA).

The V3-V4 regions of the 16S rRNA gene were amplified with the 16S_F primer 5′-(TCG TCG GCA GCG TCA GAT GTG TAT AAG AGA CAG CCT ACG GGN GGC WGC AG)-3′ and 16S_R 5′-(GTC TCG TGG GCT CGG AGA TGT GTA TAA GAG ACA GGA CTA CHV GGG TAT CTA ATC C)-3′ primers, as described in the MiSeq rRNA Amplicon Sequencing protocol (Illumina, San Diego, CA, USA). The obtained amplicon (~460 bp) was cleaned with AMPure XP magnetic beads (Beckman Coulter Inc., Miami, FL, USA).

The amplicons were amplified with Illumina Nextera XT primers to add a unique combination of primers and adapter sequences. The DNA library obtained was cleaned using 50 μL of AMPure XP magnetic beads and quantified using a Quant-iT™ PicoGreen^®^ dsDNA Assay Kit (Life Technologies Corporation, Eugene, OR, USA). The DNA library of each sample was diluted to a concentration of 4 nM. The DNA libraries from all samples were pooled before sequencing.

The Illumina MiSeq™ platform was used for sequencing according to the manufacturer’s specifications. The QIIME2 v2022.2 software was used to analyze 274 fastq files (137 paired-end fastq files) generated during sequencing [8]. Trimming was applied to the 5’ and 3’ ends of the reads according to the phred score. Subsequently, denoising, chimera detection, and joining of the reads at 99% identity were performed using the DADA2 plugin [9], thus yielding 16,448 amplicon sequence variants (ASVs) with an average length of 437 nt. The sequences were taxonomically assigned by querying the Greengenes v13.8 nucleotide sequence database and used for the construction of rooted [10] phylogenetic tree based on the de novo phylogenetic tree approach implemented in QIIME2 [11].

### 2.5. Statistical Analysis

Statistical analysis was performed using SPSS version 28.0.1.0 software (SPSS Inc., Chicago, IL, USA).

Continuous variables are reported as mean or median ± standard deviation, according to the data distribution, and categorical data are expressed as percentage. The normality of data distributions was assessed using the Shapiro–Wilk test. Student’s *t*-test for unpaired samples, Mann–Whitney test for quantitative variables, and χ2 test for categorical variables were used to test the significance of differences between groups. For all these analyses, a *p*-value < 0.05 was considered statistically significant.

### 2.6. Fecal Microbiota Analysis

Alpha- and beta-diversity analyses were conducted using R v4.0.4 with the phyloseq v1.40.0 [12] and vegan v2.6-4 packages. Alpha diversity was calculated based on the Shannon–Weiner, Chao1and Simpson Indexes. Beta diversity was calculated with the Bray–Curtis dissimilarity and the UniFrac unweighted algorithms. Before comparative statistical analyses, the ASV abundance of each sample was normalized using the cumulative sum scaling (CSS) method [13]. Multivariate (Partial Least Square Discriminant Analysis [PLS-DA]) and univariate (Kruskal–Wallis and Manney-Whitney tests) analyses were used to determine the ASVs characterizing the five groups of participants. The Receiver Operating Characteristic (ROC) curve was used to evaluate the predictive ability of the PLS-DA model. A Venn diagram was constructed to highlight the microbial markers specific to each group. 

## 3. Results

### 3.1. Anthropometric, Clinical, and Metabolic Characteristics

According to the selection criteria, of the 105 screened patients, 104 were included and provided signed informed consent. Eighteen patients were excluded because of poor adherence to the study protocol, and 86 participants completed the study protocol. These participants were divided into three groups according to the 2018 ESC classification of the pathophysiology of syncope [2]: an OH group (*n* = 24) with orthostatic syncope, an NMS group (*n* = 26) with neuromediated syncope, and a CS group (*n* = 9) with cardiological syncope. A fourth group, the US group, included participants with syncope whose presentation did not fall into any ESC guideline category (*n* = 27) [2].

Table 1 summarizes the anthropometric, clinical, and metabolic characteristics of the four groups of patients. The four groups did not differ in anthropometric data (sex, height, waist, and DBP), non-modifiable risk factors (history of diabetes and cardiovascular disease), modifiable risk factors (alcohol consumption and exercise), and metabolic parameters (glucose, eGRF, total cholesterol, LDL cholesterol, triglycerides, hemoglobin, and white blood cell count).

Compared to the CS group, the US group had lower SBP at the first visit (*p* = 0.010). These two groups also differed in the frequency of nocturnal hypertension (*p* = 0.009) and ejection fraction (*p* = 0.027). Compared to the OH group, the US group showed a higher incidence of carotid plaques (*p* = 0.035). These two groups also differed in the prevalence of smokers (*p* = 0.006). Compared to the NMS group, the US group had a higher age (*p* = 0.031), a higher incidence of hypertension history (*p* = 0.013), higher weight and BMI (*p* = 0.29 and 0.014, respectively), and greater c-IMT (*p* = 0.008). These two groups also differed in the 24 h circadian blood pressure rhythm change index (*p* = 0.003).

### 3.2. Characterization of Fecal Microbiota

With the rarefaction normalization, we obtained a total of 10,814 ASVs with a median frequency of 76,343 ASVs/sample.

**Figure 1:**
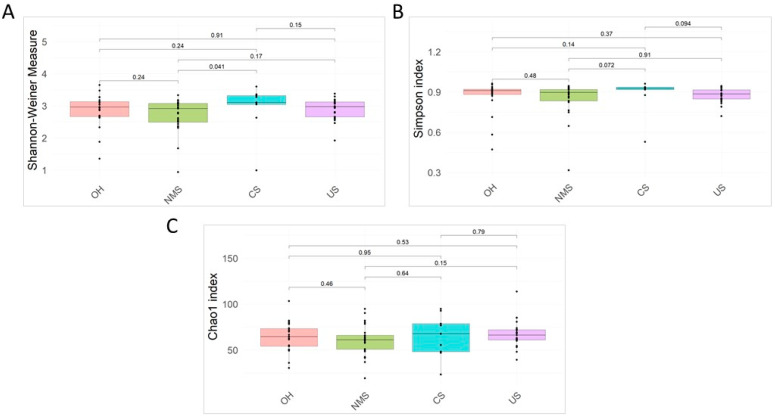
Alpha-diversity analysis. Based on the Shannon–Weiner (**A**), Simpson (**B**), and Chao1 (**C**) indexes, alpha diversity was calculated for all samples. Wilcoxon rank-sum exact test *p*-values are reported. CS: cardiological syncope; OH: orthostatic syncope; NMS: neuromediated syncope; US: unexplained syncope.

The α-diversity analysis based on the Shannon–Weiner, Simpson, and Chao1 indexes revealed a lack of evidence of a statistically significant difference among groups, except for the Shannon–Weiner index comparison between the NMS and CS groups (*p*-value = 0.041) (Figure 1).

**Figure 2:**
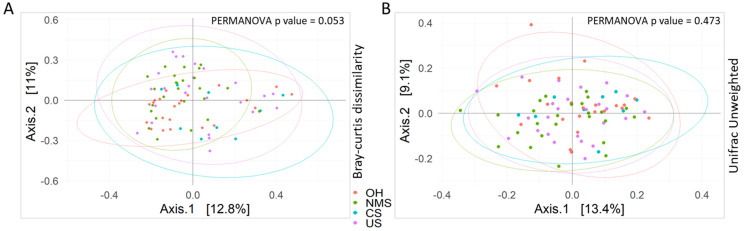
Beta-diversity analysis. Based on Bray–Curtis dissimilarity (**A**) and unweighted UniFrac (**B**) distance, beta diversity was calculated for all samples. PERMANOVA *p*-values are reported.

In addition, the PERMANOVA test applied in the β-diversity analysis, revealed the absence of intragroup distances statistically significant among all groups (*p*-value > 0.05) (Figure 2).

**Figure 3:**
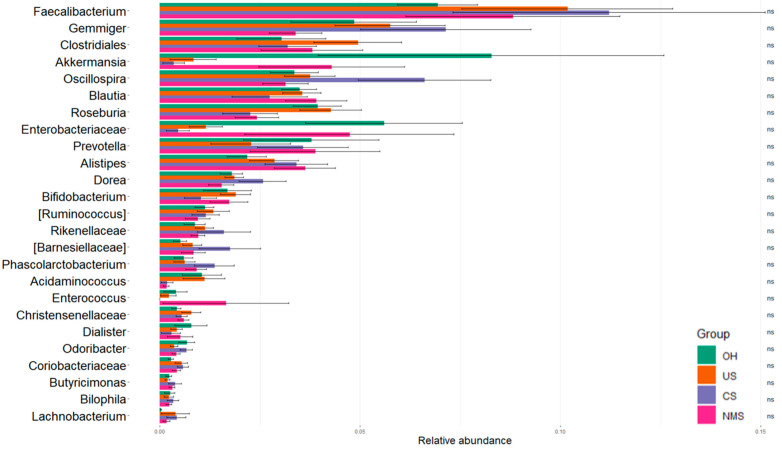
Histogram of relative abundance of ASVs at the genus level. Kruskal–Wallis test results are reported.

The uniformity of the microbiota profiles of all four groups was confirmed via univariate analysis using the Kruskal–Wallis test, which highlighted the lack of differences in the microbial composition among the four groups (*p*-value > 0.05) (Figure 3).

**Figure 4:**
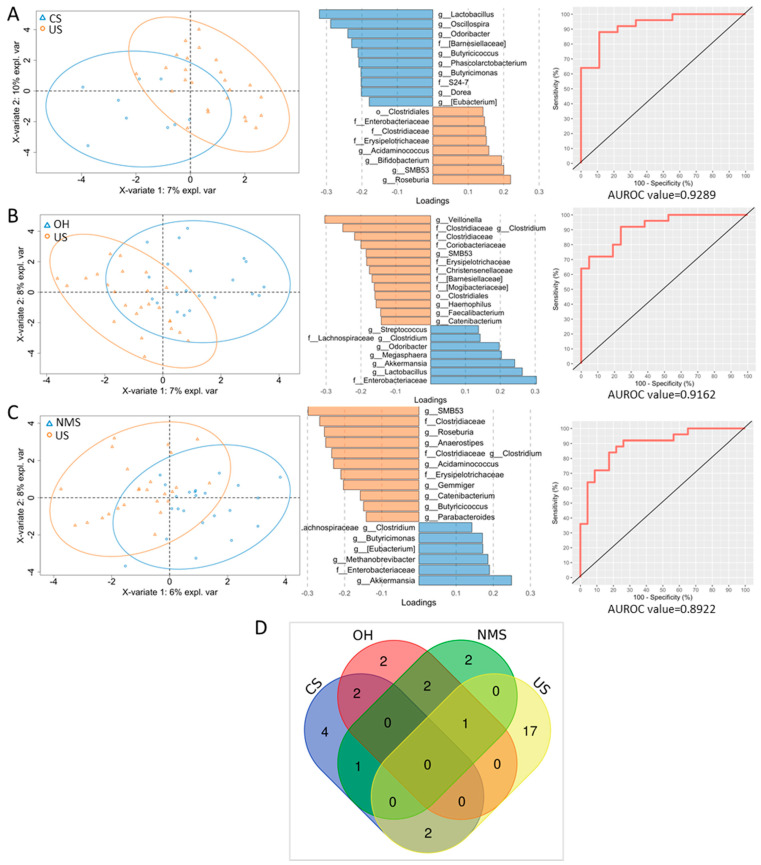
PLS-DA and loading plots show the differences in microbial taxon abundance comparing US versus CS (**A**), US versus OH (**B**), and US versus NMS (**C**). For each PLS-DA, the ROC curve with the AUROC value is reported. Venn diagram of the PLS-DA-filtered ASVs that are shared or unique amongst the TLC groups (**D**) (created by https://bioinformatics.psb.ugent.be/webtools/Venn/, (accessed on 13 December 2023)).

To test the differences between the US and other clinical classes, we performed PLS-DA (Figure 4A–C).

Interestingly, in the comparison between the US and CS groups, *Lactobacillus*, *Oscillospira*, *Odoribacter*, Barnesiellaceae, *Butyricicoccus*, *Phascolarbacterium*, S24-7, *Dorea*, and *Eubacterium* were associated with CS, whereas Clostridiales, Enterobacteriaceae, Erysipelotrichaceae *Acidaminococcus*, *Bifidobacterium*, SMB53, and *Roseburia* were associated with US (Figure 4A). In the comparison of the OH and US groups, we observed associations of *Veillonella*, *Clostridium* (Clostridiaceae), Clostridiaceae, Coriobacteriaceae, SMB53, Erysipelotrichaceae, Christensenellaceae, Barnesiellaceae, *Clostridiales*, *Haemophilus*, *Faecalibacterium*, and *Catenibacterium* with US, and associations of *Streptococcus*, *Clostridium* (Lachnospiraceae), *Odoribacter*, *Maegasphaera*, *Akkermansia*, *Lactobacillus*, and Enterobacteriaceae with OH (Figure 4B). Finally, in the comparison between the US and NMS groups, SMB53, Clostridiaceae, *Roseburia*, *Anaerostipes*, *Clostridium* (Clostridiaceae), *Acidaminococcus*, Erysipelotrichaceae, *Gemmiger*, *Catenibacterium*, *Butyricicoccus*, and *Parabacteroides* were elevated in US, and *Clostridium* (Lachnospiraceae), *Butyricimonas*, *Eubacterium*, *Methanobrevibacter*, Enterobacteriaceae, and *Akkermansia* were elevated in NMS (Figure 4C). The ROC curves confirmed the high predictive ability of the above models in assigning the samples to the right syncope class. The highest AUROC value observed in the CS versus US comparison indicated a greater difference between these two syncope classes than the difference observed in the other two comparisons.

Subsequently, the difference in the abundance of microbial taxa was assessed via a comparison of the US group versus other syncope groups using univariate analysis. The CS group had a higher abundance of *Oscillospira* and *Phascolarctobacterium* (*p* adj: 0.027) than the US group. No other differences were found in the comparisons of the US group versus the OH and NMS groups.

Combining the ASVs obtained from the PLD-DA analyses, we drew a Venn diagram to highlight the specific microbial markers of each TLC group. *Bifidobacterium*, *Clostridium* (Clostridiaceae), Erysipelotrichaceae, Clostridiales, Coriobacteriaceae, *Roseburia*, *Parabacteroides*, *Anaerostipes*, *Haemophilus*, *Veillonella*, Christensenellaceae, Clostridiaceae, *Acidaminococcus*, *Gemmiger*, *Faecalibacterium*, SMB53, and *Catenibacterium* were specifically associated with the US group; S24-7, *Dorea*, *Phascolarbacterium*, and *Oscillospira* were associated with the CS group; *Streptococcus* and *Maegasphaera* were associated with the OH group; and *Methanobrevibacter* and *Butyricimonas* were associated with the NMS group (Figure 4D, Table 2).

## 4. Discussion

Our cross-sectional pilot study was aimed at identifying the cardiometabolic characteristics of US episodes and clarifying whether US might be a subclinical manifestation of CS that must be reclassified or a different entity.

In our study sample, the US and CS groups shared most of the same anthropometric and metabolic characteristics, and most of the modifiable and non-modifiable risk factors. However, they differed in some important cardiovascular risk factors. The significantly different elements of clinical relevance in terms of cardiovascular risk factors were systolic hypertension at the first visit and the lower frequency of subjects without nocturnal hypertension during the 24 h monitoring in the CS group. The sympathetic nervous system regulates cardiovascular homeostasis by controlling BP, vascular resistance, heart rate, and cardiac contractility. Indeed, through the baroreflex, large changes in BP in response to postural changes and intravascular volume are prevented. However, the role of the baroreflex is primarily to inhibit large variations in BP throughout the day, whereas its effects on long-term BP control remain controversial [14]. The baroreflex causes rapid changes in BP, whereas continuous stimulation of the baroreceptors revealed that, after a few minutes, the baroreceptors fail to lower BP [15,16]. Furthermore, resection of carotid baroreceptors in baboons has been found to only transiently increase BP, thus suggesting that the baroreflex does not have a long-term effect on BP [17]. Therefore, the origin of hypertension in CS is not found to be associated with an alteration of the baroreflex. Regarding the 24 h trends in BP, a 10–20% decrease in physiological blood pressure occurs during the night [18]. Preservation of nocturnal dipping has a cardioprotective effect, whereas the loss of nocturnal dipping or the occurrence of an inverse pattern due to the presence of nocturnal hypertension is associated with a significantly elevated risk of developing cardiovascular diseases [19].

The US group did not show substantial clinically relevant differences, even when compared with the OH group, except for the higher incidence of carotid plaques. The greatest differences were found in the comparison of the NMS group and US group, the latter of which showed a higher incidence of risk factors for cardiovascular and metabolic diseases (age, weight, BMI, history of blood hypertension, and c-IMT thickness). These two groups also differed in the 24 h circadian blood pressure rhythm change index.

US is well known to be correlated with an elevated risk of coronary events, aortic valve stenosis, death due to cardiovascular diseases [19], and sudden cardiac death [4]. The relative risk of sudden cardiac death in patients with a recent US event (<6 months) is five times higher than that in patients without syncope. Therefore, both the American College of Cardiology Foundation/American Heart Association guidelines (class IIa) and the ESC guidelines [2,5] include US in the assessment of implantable cardioverter-defibrillator placement. US and CS might potentially share a common pathogenic basis—atherosclerosis—that differentiates them from the benign manifestations of syncope, as evidenced in our study sample. Atherosclerosis is a chronic inflammatory disease that affects primarily the vascular intima, thus leading to a greater c-IMT and the formation of plaques. Atherosclerotic plaques constitute a pathophysiological element of arrhythmias, and are cardiac or vascular structural impairments that cause CS [20]. Risk factors for cardiovascular and metabolic diseases contribute to atherosclerotic damage and related complications, thus resulting in the manifestation of the clinical presentation observed in CS [21,22]. Carotid intima–media thickening arises from a combination of intimal changes associated with atherosclerosis and medial changes due to vascular hypertrophy [23]. This thickness is a surrogate measure for subclinical atherosclerosis and is influenced by age, sex, race, and cardiovascular risk factors (e.g., obesity, hypertension, diabetes, dyslipidemia, and smoking) [24,25]. Measurement of c-IMT may be useful for identifying asymptomatic patients at a high risk of cardiovascular diseases [26]. A close correlation of c-IMT with risk factors of atherosclerosis duration and intensity has been demonstrated. Greater c-IMT, representing a subclinical phase of atherosclerotic disease, is a predictor of coronary, cerebrovascular, and peripheral arterial occlusive diseases [27]. For every 0.1 mm increase in c-IMT, the risk of heart attack increases by 11% [28]. In addition, in an apparently healthy population, the rate of estimated events that range from >5% to >10% in 10 years is 16.22%. This rate increases to 36.6% if the presence of asymptomatic carotid plaques is considered in the risk calculation [29].

Considering the differences that emerged from the comparison between the four groups, the substantially higher c-IMT in the US group compared to the NMS group, the higher incidence of carotid plaques in the US group compared to the OH group, and the absence of significant differences between the US group and the CS group in terms of predictors of atherosclerotic damage, it can be suggested that US is a preclinical manifestation of an arrhythmic or structural disease of the heart and large vessels, leading to cardiovascular events including CS. However, in our study sample, we did not observe fully concordant risk and clinical profiles between the US and CS groups. This finding prompted us to consider other biomarkers.

The possible role of the gut microbiota as a biomarker in US pathogenesis was evaluated. A study conducted by Bai et al. [6] reported a predominance of Ruminococcaceae in a sample of children with vasovagal syncope compared to healthy people, thus suggesting that the gut microbiota might be involved in vasovagal syncope development. More generally, intestinal dysbiosis might be involved in the pathophysiological mechanisms of syncope because of its connection to the central nervous system in the microbiota–gut–brain axis [6,7].

Our overall comparison of the TLC groups indicated similar microbiota profiles among the groups. However, separate comparisons of the US group versus the CS, OH, and NMS groups revealed specific bacterial biomarkers associated with each group. Notably, this approach highlighted a specific US gut microbiota profile that was differentially enriched of certain unique bacteria in comparison to other gut microbiota bacteria. In fact, we obtained 17 US-specific bacterial markers, but only four biomarkers were specifically associated with CS and two were associated with OH and NMS.

Moreover, the highest AUROC value, indicating the best model prediction, was obtained from the US/CS analysis. Thus, from a gut microbiota viewpoint, US and CS are more different from each other than US is from OH or NMS. This finding suggests the hypothesis that US and CS are not two manifestations of the same pathology, at least in terms of the microbiota.

The diagnosis of US can hide potential pitfalls; thus, extensive and repeated diagnostic steps are necessary to detect rare or potentially malignant causes of syncope, such as catecholaminergic polymorphic ventricular tachycardia [30]. Therefore, the therapeutic approach to US to date is guided by a belief that US is a form of heart disease until demonstrated otherwise. The suggested strategies are advanced or even invasive cardiological investigations. Provocative tests (e.g., head tilt, exercise testing, and electrophysiological studies) are considered in individuals at a high risk of recurrent US [31]. Some guidelines recommend the use of an implantable loop recorder (ILR) if no evidence of heart disease is found in patients with US [32,33]. In accordance with the similarity between US and CS shown in our data, 50% of patients with US have arrhythmia detected by means of an ILR [33,34]. In patients older than 65 years with ECG abnormalities (e.g., bundle branch block, fascicular block, or prolonged PR interval), bradycardia is the most plausible cause of US [34]. Among patients with mild sinus bradycardia and a history of US, those who meet certain criteria of sinus node dysfunction, as identified in an invasive electrophysiology study, after the exclusion of reflex syncope may benefit from permanent pacing [35]. Some studies have provided important insights into neuromediated reflex responses and paroxysmal atrioventricular (AV) block, thus prompting the hypothesis that these two different mechanisms lead to the same outcome [36,37]. One or more prolonged asystolic pauses, primarily due to sinus arrest, are detected through the use of an ILR in 34% of patients with recurrent US, intact ECG, no evidence of structural heart disease, and a positive or negative tilt test. In contrast, 42% of participants with US and ECG abnormalities due to bundle branch block and syncope have a recurrence associated with AV block. In addition, Brignole et al. [38] reported that paroxysmal AV block is the cause of recurrent US in patients with normal ECG and no structural heart disease. In that study, ILR monitoring indicated the presence of sudden-onset third-degree AV block in all patients. Paroxysmal AV block is caused by an intrinsic disease of the AV node conduction system, or a neuron-mediated reflex vagal response that usually requires the presence of sinus arrest or sinus bradycardia before syncopal episodes. If neither mechanism is plausible, Brignole et al. [38] hypothesized an idiopathic origin of the AV block.

Therefore, the differential diagnosis of recurrent US is very often necessary, and the identification of recurrent syncope of a probable cardiac origin is essential to prevent fatal outcomes [30]. However, this process is often insidious. Furthermore, an arrhythmic event does not always have a pathological cardiac origin and can be the manifestation of a vagal response. Thus, some episodes of US might not necessarily be reclassifiable as cardiological pathologies because they are not subtended by pathophysiological mechanisms associated with a higher cardiometabolic risk. ILRs increase the diagnostic yield and improve cost-effectiveness, but when used as an invasive diagnostic approach, they could be useless in some patients without evidence of cardiological pathologies.

The use of new biomarkers, such as those associated with the intestinal microbiota, could guide the approach to US treatment, thus avoiding invasive procedures in patients not found to have a high cardiovascular risk. However, the results of our study should be interpreted with caution. To our knowledge, apart from the study by Bai et al. [6], there are no other reports on this topic in the literature, especially on the involvement of the gut microbiota in US. Thus, no definitive conclusion can be drawn. The differences in the composition of the gut microbiota in the four types of syncope may not be connected to pathophysiological mechanisms; they may only be a bystander or a random event. To evaluate whether the gut microbiota can be used as a biomarker in the diagnosis of US, further studies are needed to highlight its causal role and explain any underlying pathophysiological mechanisms.

The small sample size is another important limitation to consider when interpreting the results. Future studies involving a larger sample size will need to confirm the observed differences.

Furthermore, the lower number of patients included in the CS group compared to the other groups should be considered a limitation. This is because patients in the Day Hospital service were at a low risk of adverse events according to the ESC risk stratification [2], while most patients with CS were not discharged from the Emergency Department.

## 5. Conclusions

Despite extensive investigations, many syncopal episodes remain unexplained, probably due to the transient nature of these events and the complexity of the underlying mechanisms. Thus, diagnosis and risk stratification are considerably difficult in these cases. The gut microbiota could be involved in the complex pathogenesis of syncope due to the close connections in the microbiota–gut–brain axis [7]. Further investigations are needed to better clarify the predictive role of the intestinal microbiota and metabolic pathways associated with TLC.

## Figures and Tables

**Table 1:** Descriptive analysis (* mean; ** median). TLC: transient loss of consciousness; CS: cardiological syncope; OH: orthostatic syncope; NMS: neuromediated syncope; US: unexplained syncope; BMI: body mass index; SBP: systolic blood pressure; DBP: diastolic blood pressure; diurnal BH: diurnal blood hypertension; nocturnal BH: nocturnal blood hypertension; EF: ejection fraction; IMT: intima–media thickness; eGFR: Estimated Glomerular Filtration Rate; HDL cholesterol: high-density lipoprotein cholesterol; LDL cholesterol: low-density lipoprotein cholesterol.

Variables	TLC Groups				*p*-Value		
	CS (*n* = 9)	OH (*n* = 24)	NMS (*n* = 26)	US (*n* = 27)	US vs. CS	US vs. OH	US vs. NMS
Age	68.9 ± 16.0 *	57.2 ± 16.2 *	53.9 ± 13.7 *	62.5 ± 14.5 *	0.270	0.225	**0.031**
Sex (M/F)	5 (5.8%)/4 (4.7%)	11 (12.8%)/13 (15.1%)	12 (14.0%)/14 (16.3%)	13 (15.1%)/14 (16.3%)	0.700	0.869	0.884
Altered sleep quality (*n*/y)	4 (4.8%)/5 (6.0%)	8 (9.6%)/16 (19.3%)	5 (6.0%)/19 (22.9%)	6 (7.2%)/20 (24.1%)	0.221	0.420	0.848
Alcohol (*n*/y)	6 (7.1%)/3 (3.5%)	19 (22.4%)/5 (5.9%)	21 (24.7%)/4 (4.7%)	19 (22.4%)/8 (9.4%)	0.835	0.472	0.244
Smoke (*n*/y)	4 (4.7%)/5 (5.8%)	21 (24.4%)/3 (3.5%)	15 (17.4%)/11 (12.8%)	14 (16.3%)/13 (15.1%)	0.700	**0.006**	0.669
Exercise (*n*/y)	4 (4.8%)/5 (6.0%)	16 (19.0%)/8 (9.5%)	16 (19.0%)/9 (10.7%)	20 (23.8%)/6 (7.1%)	0.070	0.420	0.311
History of hypertension (*n*/y)	5 (5.8%)/4 (4.7%)	14 (16.3%)/10 (11.6%)	21 (24.4%)/5 (5.8%)	13 (15.1%)/14 (16.3%)	0.700	0.467	**0.013**
History of diabetes (*n*/y)	7 (8.1%)/2 (2.3%)	21 (24.4%)/3 (3.5%)	25 (29.1%)/1 (1.2%)	22 (25.6%)/5 (5.8%)	0.808	0.555	0.092
Cardiovascular diseases (*n*/y)	7 (8.1%)/2 (2.3%)	21 (24.4%)/3 (3.5%)	24 (27.9%)/2 (2.3%)	23 (26.7%)/4 (4.7%)	0.606	0.811	0.413
History of syncope (*n*/y)	4 (4.8%)/4 (4.8%)	9 (10.8%)/14 (16.9%)	6 (7.2%)/20 (24.1%)	9 (10.8%)/17 (20.5%)	0.434	0.744	0.358
Weight	70.0 ± 13.4 **	70.0 ± 20.2 **	65.5 ± 17.2 **	78.0 ± 12.5 **	0.241	0.91	**0.029**
Height	168 ± 9.3 **	165 ± 34.7 **	170 ± 9.7 **	167 ± 9.3 **	0.783	0.520	0.852
BMI	24.6 ± 2.9 **	25.7 ± 3.4 **	24.1 ± 4.6 **	26.7 ± 4.3 **	0.125	0.385	**0.014**
Waist	91.5 ± 8.0 **	94.5 ± 11.5 **	87.0 ± 16.3 **	98.0 ± 12.3 **	0.215	0.437	0.082
SBP	145 ± 12.1 *	134 ± 14.3 *	125 ± 13.5 *	129 ± 15.7 *	**0.010**	0.303	0.340
DBP	81.9 ± 10.5 *	77.9 ± 9.2 *	78.6 ± 8.7 *	77.2 ± 10.8 *	0.226	0.807	0.608
Orthostatic hypotension (n/Y)	7 (8.5%)/2 (2.4%)	18 (22.0%)/5 (6.1%)	24 (29.3%)/2 (2.4%)	20 (24.4%)/4 (4.9%)	0.712	0.659	0.329
Diurnal BH (*n*/y)	2 (2.4%)/7 (8.5%)	15 (18.3%)/8 (9.8%)	17 (20.7%)/8 (9.8%)	15 (18.3%)/10 (12.2%)	0.052	0.709	0.515
Nocturnal BH (*n*/y)	2 (2.4%)/7 (8.5%)	16 (19.5%)/7 (8.5%)	19 (23.2%)/6 (7.3%)	18 (22.0%)/7 (8.5%)	**0.009**	0.853	0.581
Circadian rhythm of blood pressure (normal/altered)	7 (8.8%)/2 (2.5%)	13 (16.3%)/9 (11.3%)	23 (28.7%)/2 (2.5%)	13 (16.3%)/11 (13.8)	0.216	0.736	**0.003**
Circadian rhythm of heart rate (normal/altered)	4 (6.3%)/2 (3.1%)	12 (18.8%)/5 (7.8%)	12 (18.8%)/6 (9.4%)	17 (26.6%)/6 (9.4%)	0.724	0.816	0.613
EF	60.0 ± 3.3 **	62.0 ± 3.1 **	65.0 ± 2.9 **	64.0 ± 4.0 **	**0.027**	0.495	0.551
Carotid plaques (*n*/y)	1 (1.2%)/8 (9.4%)	16 (18.8%)/8 (9.4%)	14 (16.5%)/11 (12.9%)	10 (11.8%)/17 (20.0%)	0.144	**0.035**	0.171
IMT	676 ± 128 **	595 ± 114 **	555 ± 134 **	648 ± 138 **	0.689	0.075	**0.008**
Hemoglobin	14.2 ± 1.5 **	14.2 ± 2.1 **	14.1 ± 1.6 **	14.0 ± 1.0 **	0.216	0.953	0.805
White blood cells	6.4 ± 2.3 **	6.0 v 1.6 **	6.1 ± 2.2 **	6.9 ± 1.8 **	0.576	0.070	0.252
Glycemia	91.0 ± 23.4 **	97.0 ± 13.2 **	88.0 ± 11.2 **	94.5 ± 16.6 **	1.000	0.638	0.161
eGFR	80.9 ± 18.4 *	91.3 ± 15.3 *	89.3 ± 19.3 *	83.6 ± 18.6 *	0.703	0.123	0.281
Total cholesterol	192 ± 32.1 *	193 ± 33.3 *	195 ± 33.3 *	201 ± 41.7 *	0.566	0.495	0.603
HDL cholesterol	47.0 ± 21.0 **	49.5 ± 12.1 **	53.5 ± 13.1 **	52.5 ± 20.5 **	0.865	0.627	0.389
LDL cholesterol	116 ± 22.6 *	127 ± 31.0 *	124 ± 37.2 *	127 ± 44.0 *	0.510	0.993	0.815
Triglycerides	89.5 ± 43.6 **	84.0 ± 53.6 **	76.0 ± 47.2 **	102.0 ± 65.2 **	0.834	0.856	0.510

In bold and underlined values that are statistically significant.

**Table 2:** Venn diagram results. The distribution of ASVs (shared or unique) amongst the TLC groups is reported in the table. TLC: transient loss of consciousness; CS: cardiological syncope; OH: orthostatic syncope; NMS: neuromediated syncope; US: unexplained syncope; N of ASVs: number of amplicon sequence variants; ASVs: amplicon sequence variants.

TLC Groups	N of ASVs	ASVs	Phylum
NMS, OH, US	1	Enterobacteriaceae	Pseudomonadota
CS, NMS	1	*Odoribacter*	Bacteroidota
CS, OH	2	*Lactobacillus*	Bacillota
		*Eubacterium*	Bacillota
CS, US	2	*Butyricicoccus*	Bacillota
		Barnesiellaceae	Bacteroidota
NMS, OH	2	*Clostridium* (Lachnospiraceae)	Bacillota
		*Akkermansia*	Verrucomicrobiota
OH	2	*Streptococcus*	Bacillota
		*Maegasphaera*	Bacillota
NMS	2	*Butyricimonas*	Bacteroidota
		*Methanobrevibacter*	Euryarchaeota
CS	4	*Dorea*	Bacillota
		*Oscillospira*	Bacillota
		*Phascolarbacterium*	Bacillota
		S24-7	Bacteroidota
US	17	*Bifidobacterium*	Actinomycetota
		Coriobacteriaceae	Actinomycetota
		*Acidaminococcus*	Bacillota
		*Anaerostipes*	Bacillota
		*Catenibacterium*	Bacillota
		Christensenellaceae	Bacillota
		Clostridiaceae	Bacillota
		Clostridiales	Bacillota
		*Clostridium* (Clostridiaceae)	Bacillota
		Erysipelotrichaceae	Bacillota
		*Faecalibacterium*	Bacillota
		*Gemmiger*	Bacillota
		*Roseburia*	Bacillota
		SMB53	Bacillota
		*Veillonella*	Bacillota
		*Parabacteroides*	Bacteroidota
		*Haemophilus*	Pseudomonadota

## Data Availability

The source documents of the research material, in the form of paper files and electronic files, and all datasets have been deposited at the Day Hospital Service of Tor Vergata University Hospital (Rome, Italy) and are available from the corresponding author upon reasonable request, in adherence with the guidelines of *Biomedicines*.

## References

[1] Longo S., Legramante J.M., Rizza S., Federici M. (2023). Vasovagal syncope: An overview of pathophysiological mechanisms. Eur. J. Intern. Med..

[2] Brignole M., Moya A., De Lange F.J., Deharo J.C., Elliott P.M., Fanciulli A., Fedorowski A., Furlan R., Kenny R.A., Martìn A. (2018). 2018 ESC Guidelines for the diagnosis and management of syncope. Eur. Heart J..

[3] Soteriades E.S., Evans J.C., Larson M.G., Chen M.H., Chen L., Benjamin E.J., Levy D. (2002). Incidence and prognosis of syncope. N. Engl. J. Med..

[4] Hong Y., Su W.W., Li X. (2022). Risk factors of sudden cardiac death in hypertrophic cardiomyopathy. Curr. Opin. Cardiol..

[5] Goldberger Z.D., Petek B.J., Brignole M., Shen W.K., Sheldon R.S., Solbiati M., Deharo J.C., Moya A., Hamdan M.H. (2019). ACC/AHA/HRS Versus ESC Guidelines for the Diagnosis and Management of Syncope: JACC Guideline Comparison. J. Am. Coll. Cardiol..

[6] Bai W., Chen S., Tang C.S., Qi J.G., Cui Q.H., Xu M., Du J.B., Jin H.F. (2019). Gut microbiota analysis and its significance in vasovagal syncope in children. Chin. Med. J..

[7] Longo S., Rizza S., Federici M. (2023). Microbiota-gut-brain axis: Relationships among the vagus nerve, gut microbiota, obesity, and diabetes. Acta Diabetol..

[8] Bolyen E., Rideout J.R., Dillon M.R., Bokulich N.A., Abnet C.C., Al-Ghalith G.A., Alexander H., Alm E.J., Arumugam M., Asnicar F. (2019). Reproducible, interactive, scalable and extensible microbiome data science using QIIME 2. Nat. Biotechnol..

[9] Callahan B.J., McMurdie P.J., Rosen M.J., Han A.W., Johnson A.J., Holmes S.P. (2016). DADA2: High-resolution sample inference from Illumina amplicon data. Nat. Methods.

[10] DeSantis T.Z., Hugenholtz P., Larsen N., Rojas M., Brodie E.L., Keller K., Huber T., Dalevi D., Hu P., Andersen G.L. (2006). Greengenes, a chimera-checked 16S rRNA gene database and workbench compatible with ARB. Appl. Environ. Microbiol..

[11] Janssen S., McDonald D., Gonzalez A., Navas-Molina J.A., Jiang L., Xu Z.Z., Winker K., Kado D.M., Orwoll E., Manary M. (2018). Phylogenetic Placement of Exact Amplicon Sequences Improves Associations with Clinical Information. mSystems.

[12] McMurdie P.J., Holmes S. (2013). phyloseq: An R package for reproducible interactive analysis and graphics of microbiome census data. PLoS ONE.

[13] Paulson J.N., Stine O.C., Bravo H.C., Pop M. (2013). Differential abundance analysis for microbial marker-gene surveys. Nat. Methods.

[14] Fernandez G., Lee J.A., Liu L.C., Gassler J.P. (2015). The Baroreflex in Hypertension. Curr. Hypertens. Rep..

[15] McCubbin J.W., Green J.H., Page I.H. (1956). Baroceptor function in chronic renal hypertension. Circ. Res..

[16] Matton G. (1954). Carotid sinus and neurogenic and renal hypertension. J. Physiol..

[17] Bishop V.S., Haywood J.R., Shade R.E., Siegel M., Hamm C. (1986). Aortic baroreceptor deafferentation in the baboon. J. Appl. Physiol..

[18] Viggiano J., Coutinho D., Clark-Cutaia M.N., Martinez D. (2023). Effects of a high salt diet on blood pressure dipping and the implications on hypertension. Front. Neurosci..

[19] Yasa E., Ricci F., Magnusson M., Sutton R., Gallina S., Caterina R., Melander O., Fedorowski A. (2018). Cardiovascular risk after zhospitalization for unexplained syncope and orthostatic hypotension. Heart.

[20] da Silva R.M.F.L., Brugada J. (2022). Cardiac and Vascular Causes of Syncope and Atherosclerosis. Curr. Cardiol. Rep..

[21] Libby P., Buring J.E., Badimon L., Hansson G.K., Deanfield J., Bittencourt M.S., Tokgözoğlu L., Lewis E.F. (2019). Atherosclerosis. Nat. Rev. Dis. Primers.

[22] Zhu Y., Xian X., Wang Z., Bi Y., Chen Q., Han X., Tang D., Chen R. (2018). Research Progress on the Relationship between Atherosclerosis and Inflammation. Biomolecules.

[23] Wong N.D., Budoff M.J., Ferdinand K., Graham I.M., Michos E.D., Reddy T., Shapiro M.D., Toth P.P. (2022). Atherosclerotic cardiovascular disease risk assessment: An American Society for Preventive Cardiology clinical practice statement. Am. J. Prev. Cardiol..

[24] Rizza S., Longo S., Piciucchi G., Romanello D., Mavilio M., Montagna M., Coppeta L., Martelli E., Magrini A., Federici M. (2020). Carotid intimal medial thickness in rotating night shift is related to IL1β/IL6 axis. Nutr. Metab. Cardiovasc. Dis..

[25] Zoto E., Cenko F., Doci P., Rizza S. (2019). Effect of night shift work on risk of diabetes in healthy nurses in Albania. Acta Diabetol..

[26] Stein J.H., Korcarz C.E., Hurst R.T., Lonn E., Kendall C.B., Mohler E.R., Najjar S.S., Rembold C.M., Post W.S., American Society of Echocardiography Carotid Intima-Media Thickness Task Force (2008). Use of carotid ultrasound to identify subclinical vascular disease and evaluate cardiovascular disease risk: A consensus statement from the American Society of Echocardiography Carotid Intima-Media Thickness Task Force. Endorsed by the Society for Vascular Medicine. J. Am. Soc. Echocardiogr..

[27] Poredos P., Jezovnik M.K. (2021). Preclinical carotid atherosclerosis as an indicator of polyvascular disease: A narrative review. Ann. Transl. Med..

[28] Salonen J.T., Salonen R. (1991). Ultrasonographically assessed carotid morphology and the risk of coronary heart disease. Arterioscler. Thromb..

[29] Novo S., Diana D., Tomasino C., Zambelli G., Mignano A., Scalmato A., Maniscalco L., Galassi A.R., Matranga D., Novo G. (2021). Electrocardiographic abnormalities, preclinical carotid atherosclerosis and cardiovascular risk in an apparently healthy real-world population. Data from the “No Stroke, No Infarction” project of the Rotary International—District 2110 (Sicily and Malta). Int Angiol..

[30] Bergau L., Sohns C., Sommer P. (2022). Unexplained syncope in a young athlete: The diagnostic process to find the diagnosis a case report. Eur. Heart J. Case Rep..

[31] Runser L.A., Gauer R.L., Houser A. (2017). Syncope: Evaluation and Differential Diagnosis. Am. Fam. Physician.

[32] Davis S., Westby M., Pitcher D., Petkar S. (2012). Implantable loop recorders are cost-effective when used to investigate transient loss of consciousness which is either suspected to be arrhythmic or remains unexplained. Europace.

[33] Krahn A.D., Klein G.J., Yee R., Hoch J.S., Skanes A.C. (2003). Cost implications of testing strategy in patients with syncope: Randomized assessment of syncope trial. J. Am. Coll. Cardiol..

[34] Mehta N., Tavora M.Z., Morillo C.A. (2011). Explaining the unexplained causes of syncope: Are we there yet?. J. Am. Coll. Cardiol..

[35] Doundoulakis I., Gatzoulis K.A., Arsenos P., Dilaveris P., Skiadas I., Tsiachris D., Antoniou C.K., Soulaidopoulos S., Karystinos G., Pylarinou V. (2020). Permanent pacemaker implantation in unexplained syncope patients with borderline sinus bradycardia and electrophysiology study-proven sinus node disease. J. Arrhythm..

[36] Brignole M., Menozzi C., Moya A., Garcia-Civera R., Mont L., Alvarez M., Errazquin F., Beiras J., Bottoni N., Donateo P. (2001). Mechanism of syncope in patients with bundle branch block and negative electrophysiological test. Circulation.

[37] Moya A., Brignole M., Menozzi C., Garcia-Civera R., Tognarini S., Mont L., Botto G., Giada F., Cornacchia D., International Study on Syncope of Uncertain Etiology (ISSUE) Investigators (2001). Mechanism of syncope in patients with isolated syncope and in patients with tilt-positive syncope. Circulation.

[38] Brignole M., Deharo J.C., De Roy L., Menozzi C., Blommaert D., Dabiri L., Ruf J., Guieu R. (2011). Syncope due to idiopathic paroxysmal atrioventricular block: Long-term follow-up of a distinct form of atrioventricular block. J. Am. Coll. Cardiol..

